# What Are Patients Told About Innovative Surgical Procedures? A Qualitative Synthesis of 7 Case Studies in the United Kingdom

**DOI:** 10.1097/SLA.0000000000005714

**Published:** 2022-09-30

**Authors:** Daisy Elliott, Cynthia A. Ochieng, Jesmond Zahra, Angus G.K. McNair, Barry G. Main, Anni Skilton, Natalie S. Blencowe, Sian Cousins, Sangeetha Paramasivan, Christin Hoffmann, Jenny L. Donovan, Jane M. Blazeby

**Affiliations:** * Centre for Surgical Research, National Institute for Health Research Bristol and Weston Biomedical Research Centre, Surgical Innovation Theme, Population Health Sciences, Bristol Medical School, University of Bristol, Bristol, England; † Centre for Surgical Research, National Institute for Health Research Bristol and Weston Biomedical Research Centre, Surgical Innovation Theme, Population Health Sciences, Bristol Medical School, North Bristol NHS Trust, University of Bristol, Bristol, England; ‡ Centre for Surgical Research, National Institute for Health Research Bristol and Weston Biomedical Research Centre, Surgical Innovation Theme, Population Health Sciences, Bristol Medical School, University Hospitals Bristol, Weston NHS Foundation Trust, University of Bristol, Bristol, England; § Centre for Surgical Research, National Institute for Health Research Bristol and Weston Biomedical Research Centre, Surgical Innovation Theme, Population Health Sciences, Bristol Medical School, University Hospitals Bristol, Weston NHS Foundation Trust, University of Bristol, Bristol, England; ∥ Population Health Sciences, Bristol Medical School, University of Bristol, Bristol, England

**Keywords:** innovation, informed consent, surgery, qualitative

## Abstract

To investigate how information about innovative surgical procedures is communicated to patients.

**Background:**

Despite the national and international guidance that patients should be informed whether a procedure is innovative and has uncertain outcomes, little is known about current practice.

**Methods:**

This qualitative study followed 7 “case studies” of surgical innovation in hospitals across the United Kingdom. Preoperative interviews were conducted with clinician innovators (n=9), preoperative real-time consultations between clinicians and patients were audio-recorded (n=37). Patients were interviewed postoperatively (n=30). Data were synthesized using thematic analytical methods.

**Results:**

Interviews with clinicians demonstrated strong intentions to inform patients about the innovative nature of the procedure in a neutral manner, although tensions between fully informing patients and not distressing them were raised. In the consultations, only a minority of clinicians actually made explicit statements about, (1) the procedure being innovative, (2) their limited clinical experience with it, (3) the paucity of evidence, and (4) uncertainty/unknown outcomes. Discussions about risks were generalized and often did not relate to the innovative component. Instead, all clinicians optimistically presented potential benefits and many disclosed their own positive beliefs. Postoperative patient interviews revealed that many believed that the procedure was more established than it was and were unaware of the unknown risks.

**Conclusions:**

There were contradictions between clinicians’ intentions to inform patients about the uncertain outcomes of innovative and their actual discussions with patients. There is a need for communication interventions and training to support clinicians to provide transparent data and shared decision-making for innovative procedures.

Surgical innovation is key to improving patients’ quality and length of life. Although difficult to define,^1–3^ innovation exists on a spectrum; an innovative invasive procedure can be characterized as a new or modified procedure that differs from currently accepted local practice, the outcomes of which have not been fully systematically evaluated and reported in a standardized manner, and which may entail unknown outcomes to the patient.^4,5^

Guidance from the American College of Surgeons and from the UK General Medical Council on informed consent states that patients should understand the risks, as well as the benefits of the proposed operation and that information is presented fairly, clearly, and accurately and be informed if a treatment option is innovative.^6,7^ Moreover, shared decision-making (SDM) is a cornerstone of current clinical practice. Although there is no single accepted definition of SDM,^8^ this means that professionals, patients, and significant others must work together so that patients are supported to reach decisions based on evidence and informed by personal preferences, health beliefs, and values.

Some evidence has suggested fundamental deficiencies in how informed patients are before undergoing new procedures,^9^ including mesh implants for surgical treatment of urinary incontinence, which have been implanted in millions of women worldwide. Research exploring retrospective views in the United States and United Kingdom also suggests that information given to patients can vary considerably in content and quality.^10,11^ However, current practice of information provision has not yet been investigated in the context of real doctor–patient interactions.

The current study aimed to explore what information is presented to patients about innovative surgical procedures. Specific objectives were to investigate (1) clinicians’ intentions to discuss innovative procedures with patients, (2) how information was communicated to patients in consultations, and (3) patients’ views of this information in subsequent interviews.

## METHODS

### Study Design

The study protocol has previously been published.^12^ We followed “case studies” of innovation over time,^13,14^ and data sources for this study consisted of (1) “background” interviews with clinicians responsible for the introduction of the innovative procedure to generate an in-depth understanding of the procedure, including exploring what it involved, how it was innovative, accounts of evidence for the procedure, and views as to what patients should be told; (2) audio-recording consultations between clinicians and patients to understand how innovative treatments are discussed; and (3) interviews with patients to explore personal views on the presentation of information provided about the procedure during consultations, reasons underlying decisions to accept or decline the procedure, views of innovation, and (if relevant) their experience of undergoing the procedure and subsequent recovery.

The qualitative methods utilized in the current study (clinician interviews, audio-recording consultations, follow-up patient interviews) were adapted from research exploring information provision in trials.^15^ It is expected that further adaptions will develop as methods to optimize recruitment into early phase studies are more formally developed. Ethical approval was granted by the Frenchay Research Ethic Committee (Ref 18/SW/0277). The study is reported according to Standards for Reporting Qualitative Research^16^ and Reporting Involvement of Patients and the Public-2^17^ (Supplementary File 1, Supplemental Digital Content 1, http://links.lww.com/SLA/E281).

### Selection of Case Studies

Case studies were eligible if they were deemed to be an innovative invasive procedure and/or device (IP/D).^12^ A senior academic surgeon (J.M.B.) approached individuals from National Health Services (NHS) trusts (an organizational unit within England that provides health care to the local population), NHS Clinical Effectiveness Committees (which provide local governance for the oversight on invasive procedures),^18^ and funding bodies to identify innovative IP/Ds.

A background interview was conducted to learn more about the innovative IP/D. The team then reviewed existing literature to identify published articles on the procedure. Identification of new IP/Ds was initially opportunistic,^19^ although became more purposive and aimed to seek out a range of procedures to capture innovation in different contexts to achieve maximum variation.^20^ This included varying representation of procedures in relation to stages of innovation, surgical specialty, type of innovation (procedure or device), and geographical location.

### Recruitment and Sampling of Study Participants

The clinician responsible for introducing the procedure in their hospital was initially identified, and any additional key health care professionals (ie, those who also discussed the procedure with patients) were subsequently identified by the snowball technique. Health care professionals were requested to audio-record all appointments where they provided information to eligible patients about the treatment options, including the innovative IP/D, until a decision was made.

Patients were identified and approached by the surgical team to see whether they would consider taking part in the study. Patients were eligible to take part in the study if they were being offered or had recently undergone (within 3 months from discharge) an innovative IP/D. Only patients who were over 18 years old and had the capacity to consent were eligible to take part.

Research nurses shared consenting patients’ contact details with the research team once they had been discharged, with patients often specifying a time frame for initial recovery before they wished to be called by the research team for an interview.

### Data Collection

Multiple experienced qualitative researchers (D.E., C.O., J.Z., and C.H.) conducted one-to-one interviews (Supplementary File 2, Supplemental Digital Content 2, http://links.lww.com/SLA/E282), enabling investigator triangulation (having multiple researchers contribute different perspectives during data collection to add breadth to the phenomenon of interest^21^). Semistructured interviews were directed by a topic guide to ensure that the same core areas were consistently covered among the team of interviewers, while allowing flexibility to pursue the detail that was salient to each participant.^22^ These were based on previous literature and D.E.’s knowledge and experience of surgical innovation and informed consent and it was then reviewed and edited by the research team (C.O., J.Z., S.P., N.S.B., S.C., C.H., A.S., and J.M.B.) and refined as data collection/analysis progressed to enable exploration of identified findings.^23,24^ Separate topic guides for health care professional and patient interviews were developed (Supplementary File 3, Supplemental Digital Content 3, http://links.lww.com/SLA/E283). Regular team meetings allowed for the team to review the topic guide in light of findings and consider potential changes (eg, addition of topics or rephrasing of questions). All interviews and consultations were audio recorded, transcribed verbatim, and de identified.

### Data Analysis

Data were analyzed using qualitative content and thematic analysis methods based on the techniques of constant comparison and grounded theory.^25–27^ A grounded theory methodology enabled the inductive identification of themes that were derived from, or grounded in, the data. Its central principle is of constant comparison, where new findings are systematically compared with existing data so that similarities and differences can be identified through the ongoing assimilation of data.^25,28^

First, D.E. repeatedly read the background interview, each consultation and, where available, the corresponding patient interview. This initial process of familiarization with the data enabled D.E. to become immersed in the data and reflect on the words actively, analytically, and critically,^27^ reflecting upon what the clinician felt was the key information about the procedure, what the patient was told in the consultation, and how patients interpreted this information in the follow-up interview. During this process, D.E. made detailed notes about salient concepts and ideas.^29^

A coding framework was organized into a matrix in Microsoft Excel (version 2102, Microsoft) to facilitate comparisons across case studies to identify patterns and variations in how new procedures were discussed. Within this, available data from the same patient were stored in paired rows so that the consultation was next to the follow-up patient interview to enable comparisons between what was said in the consultation and the patient’s views. Data were then grouped according to the clinician leading the appointment. This enabled comparisons within a single clinician’s appointments to understand typical practices, identify patterns between other clinicians, and explore wider variation across case studies. As coding progressed, D.E. began to construct themes and subthemes to convey a meaningful embodiment of the data.^29^

D.E. was mindful to identify “negative cases” (ie, participants whose experiences differed from the main body of evidence) to enhance credibility by ensuring a wider variety of circumstances were incorporated into the findings.^25^ A portion of the data was double coded by another qualitative researcher (C.O.) and 2 academic surgeons with expertise in informed consent and SDM (A.G.K.M. and B.G.M.). It was felt that investigator triangulation—from multiple coders with differing backgrounds, research skills, and experiences—would add breadth to analysis.^30,31^

Analysis initially focused on the 2 case studies, which had yielded the largest number of recorded appointments and patient interviews. Data from 3 other case studies were subsequently analyzed to confirm, challenge, and develop themes. This was followed by analysis of 2 case studies to review these findings in relation to the existing themes,^27,29^ as a quality control measure and to reflect whether saturation had been achieved.^32^ At this stage, changes to the existing codes were minimal and mostly related to renaming the themes or reordering the subthemes to ensure they conveyed the overall story of the data sets. Analysis continued until saturation had been achieved,^32^ in that no new themes or lines of enquiry were being identified that contributed to the research question.^20^

### Patient and Public Involvement

A patient group was established, where we asked 4 individuals who had undergone surgery in the NHS to give their impressions of 2 consultations from 2 case studies (Table 1).

**TABLE 1:** Patient and Public Views on Consultation Data

Patient and public involvement in the study
Four patient/public partners were sent 2 anonymized transcripts of consultations from 2 case studies. We asked each person to reflect on their impressions of the consultations, in terms of the information presented about the new procedure and the decision-making process.
We then facilitated a 1-hour group discussion with 3 partners via video platform and organized a one to-one telephone call with a fourth partner who had been unable to join the session (because of technology issues).
From the information in the transcripts, all individuals had perceived the procedures to be established and felt that more clarity was needed about the novelty of the procedure. Terminology was felt to be too technological, and that information was not personalized to the specific patient, thus making it difficult to make an informed decision. Partners commented that given the volume of information presented was overwhelming, patients needed a written information to supplement the verbal information, as well as sufficient time to absorb and process the information, and discuss with family/friends before deciding. Partners expressed that patients’ input into the discussion had been minimal and felt that patients needed to be provided with more opportunities to ask questions and share their views.

## RESULTS

### Data Obtained

#### Case Studies

**FIGURE 1:**
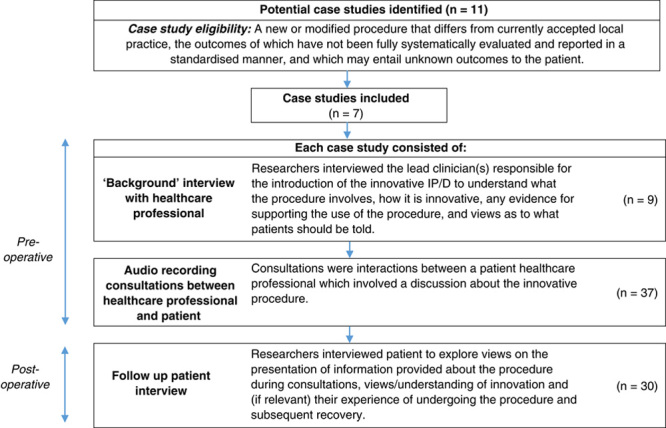
Overview of study data.

Eleven potential case studies were reviewed and 4 of these were excluded as it was agreed that they did not meet the eligibility criteria.^12^ The 7 included case studies were robotic-assisted oesophagectomy, aortic valve replacement with autologous pericardium (the Ozaki procedure), Mako robotic-assisted total hip replacement, posterior component separation for abdominal wall reconstruction, robotic-assisted Roux-en-Y gastric bypass, amniotic membrane graft for macular hole repair, and endoscopic ampullectomy. Figure 1 provides a summary of the data included.

One team had introduced the innovative IP/D as part of a research study with full Health Research Authority ethical approval, 5 had gained approvals from their hospital’s Clinical Effectiveness Committee (including 1 team that sought approval after a period of using the procedure in practice), and 1 case study did not have approval from their hospital or a research committee. In 3 cases, patients were provided with written information about the innovative component. Data were collected between August 2019 and March 2021.

### Participants

#### Background Interviews With Clinicians

Nine background interviews were conducted with clinician innovators (with at least 1 lead from each case study). Interviews lasted an average of 50 minutes (range=28–71 minutes).

#### Audio-recorded Consultations

Forty consultations were audio recorded. One patient had 2 consultations recorded, although only 1 was included in the analysis (where the innovative procedure was discussed) as the subsequent consultation covered computed tomography scan results and confirming a date for surgery. Two patients were found to be ineligible for the planned procedure during the consultation (they received the established procedure) and these data were not further analyzed nor the patients interviewed. Therefore, 37 consultations were included in the analysis. These consultations lasted an average of 16 minutes (range=7–53 minutes). In the 37 eligible patients, none declined the new procedure or requested the standard technique, although 3 patients ended up having an established procedure (the new procedure had not been available, or the clinician had made a decision not to proceed with innovative component during the operation).

As echoed by our patient advisory group, consultations tended to be led by the clinicians rather than the patients^33^ in that clinicians directed the agenda and provided information in checklist sequence before systematically attempting to elicit patient views.^34^ Recordings from the same clinician showed similarities in the content and order of information provided across consultations, suggesting consistencies in their individual communication style.

#### Patient Interviews

Interviews were conducted with 30 patients, and these lasted an average of 30 minutes (range=13–77 minutes). Of these, 9 patients had not had their consultation recorded (because of logistical reasons or them declining this aspect of the study). Although it had not been possible to obtain a recorded consultation, unpaired data (eg, conducting a patient interview without the audio-recorded consultation) still contributed to the analysis as it was felt this provided important insights. Unless there was uncertainty about the date of surgery, interviews were conducted postoperatively (35/37; 95%) to avoid influencing treatment decisions or perceptions of treatment. The average number of days between the consultation and the interview was 61 (range=16–141 days). Those who were not interviewed either declined to take part, stated they were too unwell to participate, or did not respond to the researcher. Patients’ demographics are shown in Table 2.

**TABLE 2:** Patient Demographics

Sex n (%)	
Female	24 (52)
Age (y)	
Mean (range)	62 (31–83)
Ethnicity (%)	
White British	100
Body mass index	
Mean (range)	30 (20–52)
Employment status, n (%)	
Working	25 (54)
Retired/not working	20 (44)
Student	1 (2)
Education, n (%)	
Mandated school or less	8 (17)
Higher education or more	38 (83)

### Analysis

#### Theme 1: Clinician Intentions to Discuss New Procedures (Conflicting) Descriptions About Novelty

In the background interviews, each clinician stated how the procedure was innovative. This was primarily attributed to the lack of evidence, as well as the surgeon’s own experience with the procedure and that it was not routine practice in the United Kingdom. However, as the interviews progressed, there were often tensions and conflicts in the extent the procedure was innovative, demonstrating the complexities of defining innovation.

“I say [to patients] that this is a new technique… So, to give you a simple context, there’s really only been only one report of [new procedure] in the literature, and that report has something like 9 or 10 patients… Is it hugely different to [established treatment]? Well I suppose, in a way it is… but it’s just another method of doing the same thing. Whether it’s a new procedure you know you can argue well maybe it is, maybe it isn’t… it’s probably a variation on technique or a new variation on technique” (HP5, background interview, Case Study 3).

#### Reflecting on the Challenges of Obtaining Informed Consent

In all interviews clinicians expressed a strong belief that patients should be informed of the novel status of the procedure. However, some were concerned that patients may not understand the technical complexities involved. Others queried how much information to discuss with patients and were mindful of overwhelming them, especially if some patients wanted minimal information.

“Unfortunately the patient does not have the technical element to make a really informed decision. There are people who are more suspicious and they enquire a bit more, but most of the people really take what you say […] How I convey the risk of the procedure -this is very, very difficult” (HP8, background interview, Case Study 6).

In addition, clinicians were divided as to how they perceived patients would react to innovative procedures. Some felt that patients responded very positively to new treatments but expressed concerns that they believed these would be advantageous compared with standard treatment options or they did not fully grasp what the procedure involved. Others reported that patients may be fearful of new procedures, particularly when outcomes with the existing treatment options were excellent. Overall, this suggested information provision in this context was challenging and there was a tension between fully informing patients and not distressing them.

“They’re all up for it, so if I mentioned [new procedure] a patient is like yeah, I want it. So, if it’s [new procedure], it must be better. I think it’s difficult to explain to patients that it may not be better, that we’re still learning” (HP7, background interview, Case Study 5).

“All patients are fearful of new, they want something that you’ve done lots of times, and do well, because the results here are pretty good. So, the people want to know they’re getting the same quality […] people are a bit worried when you’re starting something” (HP1, background interview, Case Study 1).

Although clinicians wanted to ensure patients were fully informed, across the consultations, there were practices that appeared to either reinforce or undermine the novelty of the new procedure, subsequently influencing patient views and expectations. These practices were not mutually exclusive, with many consultations containing statements that reinforced or undermined the innovative status of a procedure at different points. These are described below (and summarized in Table 3).

**TABLE 3:** Clinician Statements About the Innovative Procedures to Patients

		Consultations that included statement type n=37
	Type of statement	N (%)
Explaining the innovative nature of the procedure to patients	An explicit statement that the procedure is innovative	10 (27)
	Presenting the innovative treatment as “advanced” or “the future”	19 (51)
	Describing how the procedure is “the same” as established treatment	6 (16)
Explanations of experience and evidence	Clinician stating that their experience is limited	6 (16)
	Clinician explains evidence is limited	7 (19)
Presentation of risks and benefits	Clinician presents potential benefits of the procedure	37 (100)
	Clinician presents potential risks of the procedure	12 (32)
	Clinician explains there is uncertainty about outcomes	13 (35)

### Theme 2: Explaining the Innovative Nature of the Procedure to Patients

#### Explicit Statements About Novelty

Ten consultations, across 6 case studies, featured an explicit statement that the procedure was innovative (10/37 consultations). Follow-up interviews with these patients demonstrated a clear awareness that the procedure was “new.”

“It is a relatively new procedure” (Consultation 7001, Case Study 7).

“He used the words ‘fairly new’” (Patient 7001, Case Study 7).

#### A Similar—or Improved—Treatment?

Most frequently across the consultations, there were instances where the clinicians did not explicitly describe the procedure as innovative but alluded to the procedure being a different, and often improved, treatment option (19/37 consultations, across 5 case studies). This included describing the new procedure as a “progression” and the “future,” or referring to the established treatment as “old fashioned.” In the subsequent interviews, patients recalled that clinicians had been “enthusiastic” and felt the newer treatment was the “way forward.”

“You’ve gotta go to the future” (Consultation 2018, Case Study 1).

“They were enthusiastic and seemed to think it was the way forward” (Patient 2018, interview, Case Study 1).

Some clinicians also emphasized the ways that the innovative procedure was “exactly the same” to the established treatment (6/37 consultations, in 2 case studies).

“The operation itself is exactly the same” (Consultation 2020, Case Study 1).

“It’s still the same operation, we do everything the same” (Consultation 4013, Case Study 2).

### Theme 3: Explanations of Experience and Evidence

#### Descriptions of Individual and Wider Experience

In 6 consultations (6/37), across 3 case studies, the clinician stated that their experience was limited (often by explaining how many patients they had performed the procedure on). These patients subsequently recalled this in their follow-up interviews and showed a clear understanding of the procedure being innovative.

“I personally started a year ago so clearly the number of people that have been operated on this procedure is limited” (Consultation 1017, Case Study 6).

“He’s the person that does it at (names hospital), I asked is it being done in other centres, he said yes but not in every centre […] The drawback of the [new procedure] was the lack of cases” (Patient 1017, interview, Case Study 6).

In contrast, other consultations showed statements about experience that appeared to contradict the novel status of a procedure. In 3 consultations across the 3 case studies (3/37), clinicians described the extent that the innovative procedure had been used in other contexts without providing information about specific experience for the new clinical context:

“And we’ve had [new procedure] here for, I don’t know, seven, eight, nine years? And so the hospital are well used to it” (Consultation 4005, Case Study 2).

“He said he’s been doing it long enough… they are obviously all trained, he has years experience of it, so he really knows what he’s doing anyway” (Patient 4005, interview, Case Study 2).

One clinician emphasized their personal experience, the training they had undertaken and the experience of their mentor in 7 consultations, although they did not specifically explain why the training was needed.

“And I’ve done a year’s training in it and been mentored over here and signed off by my mentors who’ve done hundreds of cases” (Consultation 4006, Case Study 2).

“You’ll be all right, promise. Honestly. We do this every week, sometimes two a week. We do lots, we know what we’re doing. We’ll get you home safe, all right?” (Consultation 4009, Case Study 2).

Across the patient interviews, many appeared to believe that the procedure was more established than it was. Patients commented on the strong sense of trust they felt with their clinician, and often described feeling reassured by how confident they had appeared.

“He made me feel that he knows what he’s doing, and I’m quite happy for him to carry on” (Patient 1006, interview, Case Study 3).

“It’s an operation they do quite a lot over there, which give me a lot of confidence” (Patient 4019, interview, Case Study 2).

#### Discussing Available Evidence and Uncertainty

In the interviews, clinicians frequently described that a paucity of evidence characterized the procedure as innovative, yet this was communicated in only 7 consultations across the 3 case studies (7/37). This included informing patients about the absence of available evidence, describing what “early’” evidence was available and describing a need to generate evidence to develop an understanding of the procedure.

“We haven’t yet generated the evidence which is what part of this is, to demonstrate the evidence” (Consultation 4008, Case Study 2).

“We’re starting to collect the evidence, like with the study, to see whether that’s actually the right thing to do” (Consultation 2018, Case Study 1).

Highlighting the limited evidence appeared to convey a clear sense of uncertainty to patients. The subsequent interviews with patients suggested that they felt comfortable with this uncertainty, which was ingrained with a powerful sense of trust in their clinician.

“Clearly it’s a still a new technique that needs to be confirmed. There are lots of advantages in principle from this technique, but the longevity of this procedure needs to be confirmed” (Consultation 1017, Case Study 6).

“I’m very aware of the low numbers relative to what we know. The mind is aware. We don’t have the data, but there’s only one way of creating data. In general I’m open to newness… I mean it is actually putting my life in his hands, totally putting my life in his hands. And you know it’s a very big thing to feel the trust after one consultation. This is life and death, and I feel that I am prepared to risk it’ (Patient 1017, interview, Case Study 6).

Yet in 3 consultations across the 2 case studies (3/37), clinicians described that the evidence in a manner that implied that the procedure was not so innovative. These statements appeared to undermine the novel status of the procedure.

“From experience, but also from publications all over the world we know about the possible risks of this procedure” (Consultation 1009, Case Study 4).

“This particular procedure has been done previously, particularly in [Country], and has been reported as having good results” (Consultation 7001, Case Study 7).

### Theme 4: Presentation of Risks and Benefits

#### Clinician Investment and Beliefs

In the interviews, clinicians expressed an underlying belief that the innovative treatment represented a superior treatment option for patients than standard treatments. As the interviews progressed, it became evident that the introduction of the new procedure was facilitated by additional reasons. For instance, at an individual level, clinicians were passionate about innovation and noted their efforts to be at the forefront of surgical developments by staying up to date with trends. Some commented on a perceived demand in that patients were keen to have innovative procedures and described wider benefits to their trusts. Although many acknowledged that they were invested in the procedures, they described wanting to present an impartial and balanced perspective to patients.

“I think the main message to those patients is that we don’t have a perfect solution. I think the main message is unfortunately the solution we have has pros and cons. It is not white and black, is more an area which is grey, I think they should be informed about pros and cons of all available option” (HP8, background interview, Case Study 6).

“We say to them we don’t know whether this is going to be a benefit. We’re very open with them in saying, there might be a chance that this doesn’t confer any benefit. I’m honest with the patients. It is expensive, which is why we want to look closely, because we want to know is it cost-effective for our trust to spend money that could be spent elsewhere. Obviously I’d like that, and I love new technology and I want to use the coolest, fun thing, but we have to be sensible here, at the NHS we can’t afford it. I think we’re quite open about it” (HP1, background interview, Case Study 1).

Overall, there was evidence of a mismatch with clinicians’ intentions to inform patients about the uncertainty of the new procedure and their practice of what they said to patients. For instance, although all clinicians provided an overview of potential benefits of the innovative procedure (37/37), these advantages were often portrayed unequivocally with a sense of confidence and certainty. In addition, clinicians disclosed their own beliefs and opinions about the procedure in the 3 cases studies (18/37). The patient interviews showed that patients often picked up on their clinician’s strong beliefs.

“As a surgeon I believe it’s better… if I was here I would want the team here doing it with [new procedure]” (Consultation 4013, Case Study 2).

“I think they were more chuffed about [new procedure] than anything else at that stage…They said it had been used, it’s an improvement” (Patient 4013, interview, Case Study 2).

#### Risks Specific to the Innovative Procedure

In the background interviews, all clinicians described a range of disadvantages to the new procedure, including uncertainties and risks. In the consultations, 13 patients were informed that there was an element of “unknown” and “uncertain” outcomes for the new procedure (13/37) because of a lack of published outcome data and evidence. This was identified across 4 case studies (but only consistently mentioned in the majority of consultations for 2 cases studies; Case Studies 1 and 6).

“We’re in a grey, a very uncertain area”. (Consultation 1017, Case Study 6)

“We don’t yet know whether that has ultimately improvement of your outcomes. We don’t know that yet” (Consultation 2017, Case Study 1).

In 7 consultations, across the 2 case studies, clinicians did not mention any specific risks or unknown risks (7/37). There were also instances where clinicians explicitly stated that there were no additional risks to having the newer procedure (6/37). Although some clinicians often informed patients that the procedure took longer (22/37), only 7 went on to explain potential implications of this. Only 12 consultations from the 3 case studies described other risks or complications that were specific to the innovative procedure (12/37). Consequently, in the interviews, patients reflected mostly on the advantages of having the innovative option and when asked about potential risks were uncertain whether there were any potential disadvantages to undergoing the new procedure.

“Much of the pros and cons are unchanged by [new procedure].” (Consultation 2009, Case Study 1)

“I think the op is slightly longer […] There wasn’t any additional risks” (Patient 2009, interview, Case Study 1).

Overall, those patients who took part in the interviews commented that they had been pleased with how their surgery had gone and were satisfied with the information they received. However, several patients (particularly those who had undergone the same procedure in case study 2) reflected that their recovery had been more challenging than they had imagined and expressed concerns about whether they had fully understood the intricacies of the operation and recovery.

“I don’t believe that there is any additional risk to you having it done … We believe that it will lead to a better operation…If it all goes well your recovery is much faster” (Consultation 4012, Case Study 2).

“Obviously he must have said about the risks and whatnot but I can’t remember what was said now. Obviously [new procedure] was the better option, well the only option really … To tell you the truth I was a bit blasé about it. I thought it would be easy-peasy, know what I mean? But to be honest with you it knocked me for six the way it was, the treatment and recovery time and that sort of stuff … But I was so blasé about it. Whether I didn’t want to know anything about it, just do it, you know what I mean, they say ignorance is bliss. I just left it in their hands basically, they’re the experts, they’re the professionals, get on with it” (Patient 4012, interview, Case Study 2).

## DISCUSSION

This study used qualitative methods to explore current practice of information provision for patients undergoing novel invasive and surgical procedures in the United Kingdom. Interviews suggested that although clinicians had strong intentions to inform patients about the new status of procedures in a neutral and balanced manner, they acknowledged that communicating this information was challenging. Consultation data showed that there was variation as to whether patients were explicitly informed about the innovative status of a procedure. Furthermore, many consultations showed practices that appeared to undermine the novel status of a procedure. Although clinicians presented potential benefits of the procedure and frequently disclosed their own positive beliefs, few discussions provided an overview of potential risks and the possibility of uncertainty or unknown outcomes. Consequently, some patients were not fully aware that procedures were novel and or could have increased risks. This has important implications for informed consent.

These findings provide empirical evidence that it can be difficult for clinicians to express confidence to patients while still expressing uncertainty about the risks of novel operations.^1,35^ An inherent bias of equating newness with superiority has been identified in the literature.^36–38^ Our data showed that clinicians were extremely invested in the innovative procedures which, despite their intentions, influenced their interactions with patients. This appeared to be exacerbated by the relationship between the innovator and patient, with patients expressing a strong sense of trust in the clinician.^39^ Rogers et al^40,41^ have described how clinicians encounter specific within-role conflict of interests related to innovation that can compromise the quality of informed consent. These discussions are further complicated by the fact that with any innovative procedure, there will be inadequate data about safety and efficacy,^42^ making it difficult to disclose risks accurately to patients.^36^ Going forward, we recommend that all early phase studies report the numbers of patients offered the procedure and the number of patients who accepted/declined the procedure.

The discrepancy between clinicians’ intentions and actual practices demonstrates that obtaining informed consent for an innovative surgical procedure is a challenging process. Potential solutions include for someone other than the innovator to seek patient consent to ensure relevant information is conveyed as objectively as possible^43^ or consultation of a patient advocate.^44^ Another solution is to identify the core information domains that are required by an innovator to be discussed with a patient when offering new procedures (eg, that its effectiveness is uncertain), which is currently being developed.^45^ There is also a need to develop and evaluate interventions, which would support and train clinicians to optimize information provision and SDM. A recent Cochrane review concluded that interventions generally increased patients’ perceived knowledge and understanding, suggesting that clinicians can be trained in SDM and risk communication.^46^ Current interventions to promote informed consent generally address skills to improve how clinicians share information or direct patients to concise sources of information,^47^ although none have focused on innovative IP/Ds delivered within earlier phase studies or in clinical practice. Qualitative research can provide insights that can be used to develop interventions,^15,48,49^ and findings from the current study could begin to inform training interventions. For instance, we found that where the clinician explained how many patients they had performed the procedure on to emphasize their lack of experience, patients showed a clear understanding that there was an element of uncertainty and that procedure was innovative.

In the many countries, innovative IP/D procedures can be introduced in the context of formal research studies (eg, with Integrated Research Application System/Health Research Authority approval) or via local hospital policies, via Clinical Effectiveness Committee.^18,43,50,51^ Our study demonstrates that there was variation in how procedures had been introduced and highlights that innovative IP/Ds may also be introduced as part of routine clinical practice without formal oversight. When this occurs, there is no requirement for additional written information about the innovative component of the procedure. This risks patients not receiving full information to be able to make an informed decision. Indeed, recent research has investigated the scope of 157 NHS organization policies for the introduction of new IP/Ds into clinical practice and highlighted variation between organizations and a lack of clarity about when Research Ethics Committee (REC) application is needed.^52^ Collectively such issues underline the need to improve the oversight and regulation of information provision in this setting.^9^

Although surveys or qualitative interviews have retrospectively captured views of health care professionals,^10,11,53^ we have been unable to identify other published literature that has investigated current practice for information provision for innovative IP/Ds. Our study enabled in-depth comparisons of clinicians’ intentions and actual (rather than reported) communication practices, and patients’ perspectives of these interactions. Triangulation of multiple methods of data collection facilitated a comprehensive understanding of verbal information provision and enhanced the reliability of results.^44^

There are several limitations to note. Although we sought to identify a range of innovative procedures, only 7 case studies were selected for the study. Capturing a wider spectrum of surgical procedures would inform the wider discourse on information provision in this context. Although numbers of eligible patients are small for innovative procedures,^54^ clinicians did not systematically record all eligible consultations (as has been the case in studies audio-recording RCT discussions^55^), resulting in only a small number of recordings being obtained for some case studies. This may mean that the selected sample is not representative; however, we were still able to identify clear patterns within the data obtained across clinicians and case studies, and using unpaired data provided important insights into information provision. Moreover, where we obtained multiple recordings from the same clinician, we found that discussions were mostly led by the clinician (rather than patient led interactions^33^ and there were many similarities in what they said to patients across consultations. As the self-selected clinicians who agreed to be interviewed and to have their appointments audio recorded may have an interest or better understanding of requirements for information provision for patients,^56^ observations may not be representative of standard practice. It is important to note that only discussions about innovative procedures were captured so we cannot determine how these interactions compare with discussions about established procedures. In addition, because of the snowball sampling applied, clinicians may have been more likely to suggest colleagues who had similar views and/or communication styles. Nonetheless, we were able to capture a range of views and practices, and explored negative cases that contradicted with findings to enhance the validity of the results.^25^ Having the consultations audio recorded may have resulted in the clinicians altering their practice.^57^ However, the implication may be that in clinical practice, patients receive even less information than observed in this study.^58^

Similar to other studies that have recorded consultations and subsequently interviewed patients, the time lag between data collection points may have caused potential recall bias.^59,60^ Even with longer periods between data collection timepoints patients’ accounts matched information provided in consultations, and the interviews provided important insights into what patients felt was important. Future research should capture patient views at different timepoints, including between the consultation and surgery, to understand whether the timing of the interview affects responses and views. Preoperative interviews with patients would also provide an important perspective in this context, and could reduce the likelihood of potential issues with recall bias. Furthermore, a proportion of patients who declined to take part in interviews stated they were too unwell to participate. These patients’ experiences may have differed to those interviewed. For instance, recalled experience of information provision is quite likely to be different in those patients who have poor outcomes. As all patients interviewed were Caucasian, there is a need to recruit patients from other ethnicities to enhance the transferability of the findings. Finally, although this study provides important and rich insights into practices in the United Kingdom, future research should explore information provision in different countries and health care systems.

In conclusion, our study highlights that clinicians can find it challenging to discuss innovative invasive procedures with patients. This suggests a need to develop support and training interventions to optimize the quality of information provision and SDM in this context.

## ACKNOWLEDGMENTS

The authors thank the Lotus patients for participating in the study and the patient advisory group for the helpful feedback on the consultation data.

## Supplementary Material
