# Supplementary material for: What Are Patients Told About Innovative Surgical Procedures? A Qualitative Synthesis of 7 Case Studies in the United Kingdom
Source: Ann Surg. 2022 Sep 30;278(3):e482–90. doi: 10.1097/SLA.0000000000005714 (PMC10414150; doi:10.1097/SLA.0000000000005714)
Supplement: Supplementary file 2 [file sla-278-e482-s002.docx]

**Researcher profiles**

**Data collection**

All individuals who conducted the interviews (DE, JZ, CO, CH) are experienced qualitative researchers. All researchers are based at the University of Bristol, and each has PhDs in health-related fields using qualitative methods. DE, CO and CH are members of the [Centre for Surgical Research](https://www.bristol.ac.uk/population-health-sciences/centres/surgical-research/) (which aims to improve the surgical evidence base, and subsequently patient care, through high quality multidisciplinary research). [DE](https://research-information.bris.ac.uk/en/persons/daisy-elliott) is a Research Fellow in Qualitative Methodology Research, and at the time of interviews, [CO](https://www.bristol.ac.uk/people/person/Cynthia-Ochieng-fba2f999-9163-4c38-bf1d-f6d4b024bc96), JZ and [CH](https://research-information.bris.ac.uk/en/persons/christin-hoffmann) were Senior Research Associates in Health Services Research.

**Data analysis**

DE is a Research Fellow in qualitative research and has a PhD in Health Psychology. She is a member of the [QuinteT](https://www.bristol.ac.uk/population-health-sciences/research/groups/social-sciences-health/quintet/) research group, which uses qualitative research methods to optimise recruitment and informed consent to RCT). DE broadly identifies as a critical realist and believes that a continuum exists between positivism and relativism (1, 2). Ontologically, this proposes that truth exists and can be discovered independently of those who observe it, although these truths will be shaped by an individuals’ subjective experience, interpretation and knowledge (3, 4).

**References**

1. Robson C. Real world research: A resource for social scientists and practitioner-researchers: Wiley-Blackwell; 2002.

2. Blaikie N. Approaches to social enquiry: Advancing knowledge: Polity; 2007.

3. Braun V, Clarke V. Successful Qualitative Research: Sage; 2013 April 5th, 2013. 400 p.

4. Bhaskar R, Danermark BJSJoDR. Metatheory, interdisciplinarity and disability research: a critical realist perspective. 2006;8(4):278-97.
