# Supplementary material for: What Are Patients Told About Innovative Surgical Procedures? A Qualitative Synthesis of 7 Case Studies in the United Kingdom
Source: Ann Surg. 2022 Sep 30;278(3):e482–90. doi: 10.1097/SLA.0000000000005714 (PMC10414150; doi:10.1097/SLA.0000000000005714)
Supplement: Supplementary file 3 [file sla-278-e482-s003.docx]

**Topic guide for ‘Background’ Interviews with Healthcare Professionals**

- **Thank you** for taking the time to meet
- **Intro** – quali researcher (not HP – apologies if some questions seem silly).
- **Purpose of interview** - want to understand more about the procedure
- **Check consent form signed and happy to record** - can I just check again that you are happy for me to record the conversation?

***Would be great to understand a bit about your professional background?*** *(Current role, where based and how long)*

***As I said, I’m not a medical professional. Can you tell me more about the procedure?*** *What involves?*

- Why do you think it’s needed?
- How/when did this come about? *(Who involved?)*
- Would you describe this procedure as novel/innovative? *(Why?)*
- What patients are eligible for the procedure? *(How decided? Any reservations about particular subgroups?)*
- What other treatment options do patients have?
- Can you describe patient pathway? *(Diagnosis, information provision, eligibility assessment, operation, recovery)*
- Is there any evidence you are aware of relating to the procedure?
- What are the anticipated risks and benefits of the procedure? (*How calculated?* *Any uncertainties?)*

***Can you describe, in your own mind, the main stages of the operation?***

***What experience do you have with the procedure?*** (*If not yet* *performed*, *can you describe plans?)*

- When did you first perform the procedure? *(How many times performed? How feel when first performing?)*
- Can you describe learning process?
- Do you know of any others doing it?
- What, so far, has been the response to this procedure from your peers? *(Probe for support, opposing views)*
- We understand that more surgeons in the team will now be performing the procedure, how has that come about? Has anything been done differently in terms of preparing them, compared with when you initially started?

***In your opinion, what should patients be told about the procedure?***

- Ideally, what are the key elements that patients need to be made aware of? And how does this work in practice? *(Probe evidence, newness, training, experience)*
- What are patients’ reactions to the procedure? *(Questions, concerns, why might decline?)*
- Do you think your informed consent discussions will differ to those for standard treatments? If so how? If you don’t, why? *(Specific challenges to introducing new procedures?)*
- How long do you think patients need (or should ideally be given) to decide about the treatment?
- Has any information about procedure changed since began performing? *(If you had complications during surgery would you discuss these with your next patient? Why/why not?)*
- At what point do you think you will stop telling patients it’s new? *(How determined - number of cases, confidence?)*
- Any other HPs discussing the procedure?
- Is any written information being provided? *(When? How developed? Reviewed? - NPC, lay persons?)*
- If a patient wanted more information about the procedure, what would you say? *(Any other resources?)*
- Can you describe if any approvals required in Trust? *(Initial approval, monitoring)*

***Ending the interview***

- Thank for time
- Summarise key info
- Check additional people to interview
- Discuss logistics of recordings

**Topic guide for patients**

- **Thank you** for taking the time to talk to me.
- **Intro** - Qualitative researcher at UoB
- Purpose of interview - like to understand the kind **of information of patients that receive** about their treatments, and how they make **decisions** in healthcare.
- **Check consent form signed and happy to record** - can I just check again that you are happy for me to record the conversation?

**So I had noted down that you had had a xxx.**

- When did you have the procedure?
- How are you doing?
- Can you talk me through what the procedure involves? *[unpick where bits of info came from]*

**Want to understand a bit more about the information you received about the procedure. Can you tell me about when it was decided you would have surgery?**

- Can you recall when the surgery was first discussed with you? When? Who was this with?
- Subsequent discussion(s)? When? Who?
- When you first heard about the procedure what were your initial thoughts?
- What were you told about the risks of the operation?
- My (limited) understanding of the procedure is that it can be a variety of different ways – open, keyhole, or robot. Did you discuss which you would have? Would you have liked to? Preference? Why? Any downsides/concerns?
- Did your surgeon discuss his/her experience with the surgery? If yes, how did this make you feel? If no, do you think you would want this information?
- What were you told about recovery? In hospital/once discharged?
- Did you have any questions/concerns?
- Was there anything you felt uncertain about? *If yes*, did you address this? How?
- Did you feel there was enough information about the surgery?
- How much time were you given to think about whether you wanted to have the procedure? Was this sufficient? (probe answer)

**Besides surgery, where there any other options for you?** **Can you tell me a bit more about these?**

- Was selecting your treatment an easy or hard decision to make? (probe answer)

**What, if any, advice did you seek when considering your treatment options?**

- The internet?
- Written information? [who gave/what said/was it sufficient/easy to understand/how improved?]
- Discussion with family/friends?

**I’d like to chat you about your recovery. How do you feel the surgery went?**

- Before the surgery, what was your biggest concern?
- Would you have liked to have known anything else beforehand?
- Do you feel the outcomes you expected from the surgery have been realised? (probe if concerns mentioned earlier alleviated and for satisfaction after surgery)

**Ending the interview**

- Is there anything else you think is important to discuss?
- Any questions for me?

The next few questions are more specific and about you personally, if you feel uncomfortable answering any of these then you do not have to.
